# Effect of Low-Frequency Electrical Stimulation Combined with Tonifying Kidney and Blood Pills on Uterine Rejuvenation after Abortion

**DOI:** 10.1155/2022/9976063

**Published:** 2022-10-17

**Authors:** Man Chen, Shi-Xin Lin, Jun Zhu, Xin-Tong Chen, Ai-Hua Tan, Jun Gao, Hong Zhu

**Affiliations:** ^1^Department of Obstetrics and Gynecology, The Third Affiliated Hospital of Nanchang University, Nanchang First Hospital, Nanchang University, Nanchang, Jiangxi 330006, China; ^2^Department of Obstetrics and Gynecology, Huanggang Traditional Chinese Medicine Hospital, Huanggang, Hubei 438000, China; ^3^School of Clinical Medicine, Nanchang University, Nanchang, Jiangxi 330006, China; ^4^Department of Obstetrics and Gynecology, Jiangxi Cancer Hospital, Nanchang, Jiangxi 330029, China

## Abstract

**Objective:**

This study aimed to observe the clinical efficacy of low-frequency electrical stimulation therapy combined with tonifying the kidney and activating blood pills to promote uterine recovery after abortion and its effect on heat-shock protein (HSP)70 and HSP90.

**Methods:**

All cases were women with early pregnancy who underwent an abortion at the Third Affiliated Hospital of Nanchang University from September 2019 to September 2020. They were divided into two groups in accordance with the principle of patient voluntariness: 237 cases in the experimental group and 143 cases in the control group. Patients in both groups were given low-frequency electrical stimulation after surgery. In addition, patients in the experimental group began to take the Dingkun pill orally (one pill per time, two times per day) from the first day of surgery and continued to take it until their menstruation returned to normal. Abdominal pain, the duration of vaginal bleeding, and the amount of bleeding were observed in both groups. Uterine size, endometrial thickness, and urinary human chorionic gonadotropin (HCG) status were also observed at 2 weeks postoperatively to determine preoperative and postoperative HSP70 and HSP90 serum levels. The time of menstrual resumption, menstrual period, and menstrual volume were observed and compared with preoperative menstruation. The occurrence of complications, such as a residual uterine cavity, uterine effusion, menstrual irregularities, and reproductive tract infections, during the follow-up period was also recorded in both groups.

**Results:**

Comparison of the endometrial thickness (mm) and uterine size (sum of the three diameters) on uterine adnexal ultrasound at 2-week postoperative review was better in the experimental group than in the control group (*p* < 0.05). No statistically significant difference was found between the two groups in terms of residual uterine cavity and blood accumulation in the uterine cavity and the results of the urine pregnancy test (*p* > 0.05). Serum HSP70 and HSP90 levels were significantly higher in the control group than in the experimental group 2 weeks after surgery (*p* < 0.05). Postoperative HSP70 and HSP90 levels were significantly higher than preoperative levels in both groups (*p* < 0.05). The degree of postoperative abdominal pain in the experimental group was less severe than that in the control group, and the duration was shorter (*p* < 0.05). No statistically significant differences were observed when comparing the duration of postoperative vaginal bleeding and the amount of bleeding (*p* > 0.05). The time of the first menstrual resumption and menstrual volume were more satisfactory in the experimental group than in the control group (*p* < 0.05). No adverse reactions occurred in either group of patients receiving treatment.

**Conclusion:**

Low-frequency electrical stimulation combined with tonifying the kidney and invigorating blood pills could effectively promote uterine rejuvenation after abortion, conducive to improving patients' postoperative abdominal pain, promoting menstrual recovery and maintaining menstrual flow.

## 1. Introduction

Induced abortion is an important means of terminating unplanned and pathological pregnancies. It has the advantages of simple operation and high safety factors, and it is easily accepted by clinicians and patients. However, failure to take care of the postabortion period or multiple procedures could lead to various complications, such as prolonged vaginal bleeding, lower abdominal pain, lumbago, menstrual disorders, reduced menstrual flow, and other signs of poor uterine rejuvenation. In severe cases, it could lead to uterine and cervical adhesions, amenorrhea, tubal inflammation, and even infertility. It causes many physical and psychological injuries to most patients who have had abortions. Based on the national situation in China, reducing the damage of abortion to women when abortion is unavoidable is of great importance.

Low-frequency electrical stimulation therapy has the effect of promoting cellular metabolism and accelerating blood circulation, providing better relief for acute and chronic pain, relaxing and restoring the normal contraction and diastole of strained muscle tissue, and enhancing immunity [1]. It has a modifying and therapeutic effect on the diseases of multiple systems, including the nervous system, cardiovascular system, and respiratory system [2]. It has been widely used in obstetrics and gynecology clinics recently. Dingkun pill, the “holy medicine of the palace” has the functions of nourishing qi, blood, and yin and yang; tonifying the kidney and activating blood, regulating temperature; and eliminating dampness. It is widely used in modern medicine to treat polycystic ovary syndrome, oligomenorrhea after induced abortion, premature ovarian failure, perimenopause syndrome, postpartum uterine involution, and other diseases that have achieved good results. Some studies have shown that the Dingkun pill can effectively increase the thickness of the endometrium and prevent secondary adhesion in patients with uterine adhesion. It could significantly increase the level of endogenous sex hormones, increase the volume and duration of menstruation [3], effectively relieve abdominal pain, promote ovulation to improve menstrual irregularities, and promote uterine repair, thus reducing postoperative complications [4].

In this study, low-frequency electrical stimulation combined with tonifying kidney and an activating blood pill (Dingkun pill) was used to treat patients after abortion, and its clinical efficacy and effect on heat shock protein 70 and heat shock protein 90 (HSP70 and HSP90) in patient serum were observed. This study aimed to provide a reference and an experimental basis for the selection of methods to promote uterine rejuvenation after abortion.

## 2. Materials and Methods

### 2.1. Diagnostic Criteria

#### 2.1.1. Western Medical Diagnostic Criteria

The surgical method refers to the surgical methods of early abortion (negative pressure aspiration and forceps scraping) in the chapter on remedies for contraceptive failure in the ninth edition of Obstetrics and Gynecology published by the People's Health Press, edited by Professor Xing and Kong [5]. Early surgical abortion is mainly applied to women within 3 months of pregnancy, and in this study, abortion surgery refers to early intrauterine pregnancy up to and including 10 weeks and termination of pregnancy by negative pressure aspiration.

#### 2.1.2. Diagnostic Criteria in Chinese Medicine

Postabortion related diseases have no separate name or type of disease in Traditional Chinese medicine (TCM), but they could be treated in accordance with the symptoms of postabortion complications by referring to “postpartum diseases” [6] in the 10th edition of Chinese Medicine Gynecology, edited by Prof. Tan Yong, published by the Chinese Medicine Publishing House. “Postpartum diseases” refer to postabortion diseases caused by injuries to the maternal golden blade during pregnancy.

### 2.2. Case Criteria

#### 2.2.1. Inclusion Criteria

The inclusion criteria are as follows: (1) healthy women with early intrauterine pregnancy diagnosed with menopause ≤10 weeks or those who voluntarily requested abortion; (2) with a regular menstrual cycle in the past 6 months; (3) no contraindications to surgery and able to tolerate abortion surgery and anesthesia; (4) normal preoperative laboratory tests; (5) successful surgery and seen chorionic blastocyst and meconium tissue; and (6) patients and family members who volunteered to participate in this study and signed the informed consent form.

#### 2.2.2. Exclusion Criteria

The exclusion criteria are as follows: (1) those who did not meet the inclusion criteria; (2) with a body temperature >37.5 °C before surgery; (3) with intraoperative hemorrhage, missed aspiration, and uterine perforation; (4) with a history of sex hormone administration or uterine cavity operation within 3 months before surgery; (5) allergic to clindamycin or with contraindications related to anesthesia or oral Chinese medicine; and (6) those who could not receive regular follow-up.

#### 2.2.3. Criteria for Rejection and Detachment Cases

The criteria for rejection and detachment cases are as follows: (1) those with atypical postoperative chorionic villi or pathological results suggestive of staphylocytosis; (2) those without any remission of comorbidities or even serious adverse reactions during drug administration; (3) ultrasonography at the follow-up examination reveals excessive residual uterine tissue requiring secondary clearance or other treatment; (4) those with poor compliance and are not taking the drug according to the treatment regimen, thus affecting the efficacy, or not reviewing of time, which is not conducive to the observation; (5) those who request to withdraw from the test or miss the visit for various reasons; and (6) those who are pregnant again.

### 2.3. General Information

**Figure 1:**
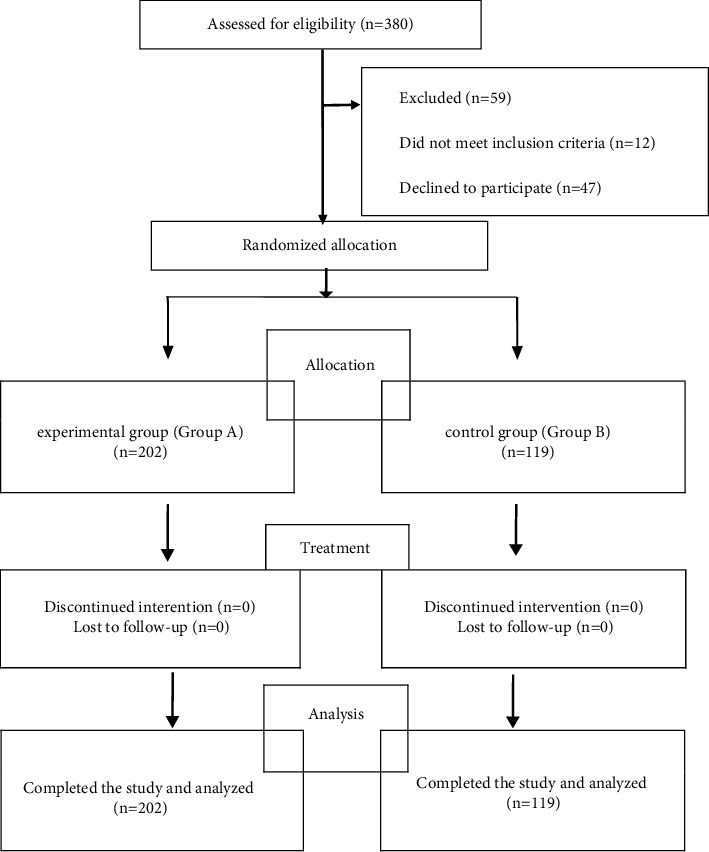
CONSORT study flow diagram.

A total of 380 enrolled cases were women with early pregnancy who underwent a painless abortion at the Third Affiliated Hospital of Nanchang University between September 2019 and September 2020. These women were divided into two groups on the basis of the principle of patient voluntariness. The actual number of cases enrolled in the experimental group was 237, and 143 cases were enrolled in the control group. A total of 202 and 119 cases were included in the experimental and control groups in strict accordance with the rejection and shedding criteria (Figure 1). No statistical difference was found in the general data of the two groups, including age, gestational age, menstrual cycle, menstrual period, number of pregnancies/births/abortions, gestational sac size, uterine size, and preoperative serum HSP70 and HSP90 concentrations (*p* > 0.05). The study was reviewed and approved by the Ethics Committee of the Third Affiliated Hospital of Nanchang University (Ethics No. KY2020034).

### 2.4. Blinding

This study was a prospective, double-blind (investigator and outcome assessor) unequal randomized controlled trial. The personnel who entered the trial data were neither involved in the process of collecting cases nor in the process of assessing the relevant indicators; the specifics of the subgroups were concealed, i.e., the interventions taken by the two groups were not directly reflected in their names, but were referred to by “Group A” and “Group B” were used to refer to them; the person who assessed the observation indicators and those who summarized and analyzed the data were blinded.

### 2.5. Treatments

(1) Postoperative infection prophylaxis (clindamycin palm dispersible tablet hydrochloride, 2–4 tablets/time, 4 times/day) and low-frequency electrical stimulation were given to both groups. (2) For low-frequency electrical stimulation treatment, a circular electrode piece was applied to the left and right of the umbilicus to stimulate the acupoints of Guan Yuan (RN4), Zhong Ji (RN3), and Uterus (EX-CA1). Then, an electrode piece was applied to the left and right of the spinous process of the second lumbar vertebra at 1.5 cm on the left and right side to stimulate the Shenshu point (BL23). The set parameters were as follows: analgesic frequency pulse (40 Hz/250 *μ*s), uterine repair frequency/pulse (40 Hz/320 *μ*s, 80 Hz/280 *μ*s, and 40 Hz/320 *μ*s), current intensity (120 mA), and time control (30 min/time). The treatment started on the day after the abortion, 30 min per treatment, and once a day, five times (a course of treatment) in total. (3) The observation group was given the supplement kidney and blood pill (one pill/time, two times/day) on top of the control group from the first day of surgery and continued to take it until the menstruation returned to normal.

### 2.6. Observation Indicators

1. Postoperative condition: The duration of postoperative abdominal pain and vaginal bleeding, the amount of vaginal bleeding, and abdominal pain were observed in both groups.
2. Menstrual recovery: The time of the return of menstruation, menstrual cycle after the resumption of natural menstruation, menstrual volume, and comparison with preoperative menstrual volume were recorded.
3. The endometrial thickness and uterine size (some of the three uterine diameters) at 2 weeks postoperation were observed.
4. HSP70 and HSP90 concentrations in serum before and 2 weeks after surgery and urine pregnancy test at 2 weeks after surgery: For the determination of HSP70 and HSP90, venous blood was collected and centrifuged at 3500 r/min for 10 min, and then the supernatant was collected and stored in a refrigerator at −80°C. The enzyme-linked immunosorbent assay (ELISA) was conducted following the instructions of the enzyme-linked ELISA test kit (Uscn).
5. Surgical complications: the occurrence of complications, such as uterine residue, uterine effusion, irregular menstruation, and reproductive tract infection during the follow-up period was recorded in both groups.

### 2.7. Statistical Analysis

The SPSS Statistics 26.0 (IBM, USA) and Prism 9.0 (GraphPad, USA) software were used to analyze the data. Enumeration data were expressed as a rate (%), and the Chi-square test was adopted. *T*-test was used for continuous variables when they obeyed the normal distribution and the Mann-Whitney U test was used when they did not. A *p* value <0.05 was taken as statistically significant.

## 3. Results

### 3.1. Establishment of Serum HSP Standard Curve

**Figure 2:**
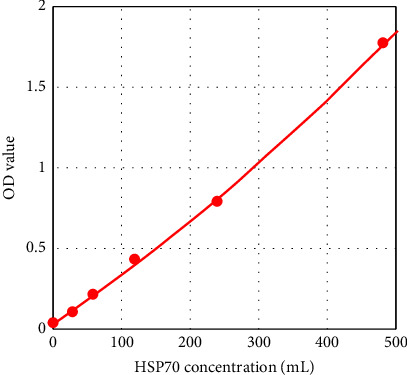
HSP70 standard curve. Regression equation *y* = 0.04202 + 0.00284 ∗ *x*(*r*^2^ = 0.99927).

**Figure 3:**
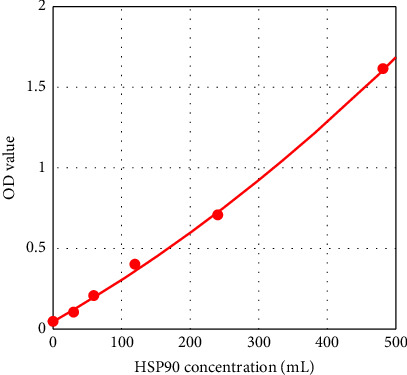
HSP90 standard curve. Regression equation *y* = 0.05078 + 0.00242 ∗ *x*(*r*^2^ = 0.99867).

The standard curve was plotted by Elisacalc software, with the concentration of the standard as the horizontal coordinate and the corresponding optical density (OD) value as the vertical coordinate (Figures 2 and 3), and the linear regression equation of the standard curve was calculated.

### 3.2. Comparison of the Degree of Postoperative Abdominal Pain

Patients in the observation group had significantly less abdominal pain than the control group, and the difference was statistically significant (*p* < 0.05), as shown in Table 1.

### 3.3. Comparison of the Duration of Postoperative Abdominal Pain and Vaginal Bleeding

The duration of abdominal pain in the observation group was significantly shorter than that in the control group, and the difference was statistically significant (*p* < 0.05). No statistically significant difference could be found in the duration of vaginal bleeding between the two groups (*p* > 0.05), as shown in Table 2.

### 3.4. Comparison of Postoperative Vaginal Bleeding

No statistically significant difference could be found in the amount of postoperative vaginal bleeding (mL) between the two groups (*p* > 0.05), as shown in Table 3.

### 3.5. Review of Ultrasound after 2 Weeks

**Figure 4:**
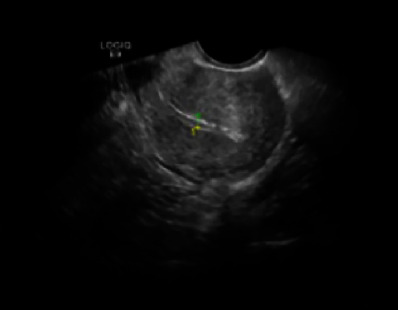
The endometrial line was clear, the thickness of the endometrium was 6 mm, and no residual pregnancy or fluid was present in the uterine cavity.

**Figure 5:**
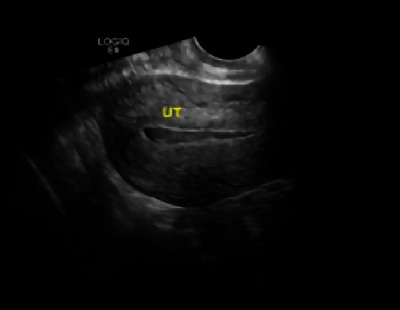
The UT (uterine) cavity was separated, and a small amount of fluid was seen in the uterine cavity at approximately 4 mL.

When comparing the endometrial thickness (mm) and uterine size (sum of three diameters), a significant difference was found between the two groups (*p* < 0.05, Table 4). The difference was not statistically significant (*p* > 0.05) when the uterine cavity residue and uterine cavity blood accumulation were compared (Table 5). Ultrasound images of the randomly selected experimental group for review are shown in Figure 4. The control group for review is shown in Figure 5.

### 3.6. Comparison of Urine Pregnancy Test Results at 2 Weeks Postoperation

No statistically significant difference (*p* > 0.05) was found in the comparison of the results of the urine pregnancy test at 2 weeks after surgery between the two groups, as shown in Table 6.

### 3.7. Comparison of Preoperative and Postoperative Serum HSP70 and HSP90 Concentrations (pg/mL)

No statistically significant difference could be observed in the preoperative serum levels of HSP70 and HSP90 (pg/mL) between the two groups (*p* > 0.05). Two weeks after surgery, the serum HSP70 and HSP90 concentrations were significantly higher in both groups compared with those before surgery, and the difference was statistically significant (*p* < 0.05). Higher postoperative serum HSP70 and HSP90 concentrations were found in the control group than in the observation group, and the differences were statistically significant (*p* < 0.05), as shown in Table 7.

### 3.8. Comparison of Menstrual Recovery

A statistically significant difference (*p* < 0.05) was observed in the time to return to menstruation and the period of menstruation after surgery between the two groups, as shown in Table 8. The observation group had statistically significant differences (*p* < 0.05) when comparing the period of return to menstruation with the preoperative period, but no statistically significant difference (*p* > 0.05) was found between the period of return of menstruation in the control group and the preoperative period, as shown in Table 9. The difference was statistically significant (*p* < 0.05) when comparing the first resumption of menstruation between the two groups, as shown in Table 10. The findings indicated that the treatment regimen in the observation group was effective in promoting the resumption of menstruation.

### 3.9. Comparison of Postoperative Adverse Drug Reactions

No postoperative adverse drug reactions occurred in the experimental and control groups.

## 4. Discussion

Abortion is the basic measure for terminating early unplanned and pathological pregnancies. Although the operation is simple and quick, the trauma it causes the human body is undeniable. Improper operation and postoperative care often lead to complications, such as lower abdominal pain, prolonged vaginal bleeding, residual uterine cavity, uterine adhesions, pelvic inflammatory disease, menstrual disorders, and decreased menstrual flow. Western medicine believes that uterine rejuvenation is mainly manifested by the contraction of uterine muscle fibers and the repair and regeneration of the endometrium. Therefore, for patients who undergo abortions, oxytocin is often promoted with contraction hormones to expel the residual pregnancy tissue and reduce vaginal bleeding. For patients with poor endometrial growth and endocrine disorders, an artificial cycle is established with combined estrogen and progestin therapy; otherwise, oral contraceptives, such as mafron, eosin, and daing 35, are used to accelerate endometrial repair to prevent uterine adhesions and reduce abdominal pain [7]. However, its efficacy is not good in the long term. No record of abortion could be found in Chinese medicine. By observing the postoperative clinical manifestations of patients who underwent abortion and the damage caused to women's bodies by abortion, modern Chinese medicine practitioners have classified it as “abortion,” “miscarriage,” and “postpartum disease” [8]. According to Chinese medicine, “the kidneys store sperm, which is the main source of reproduction,” and the combination of sperm from both sexes is the key to the formation of the fertilized egg. After the fertilized egg is formed, the qi and blood of the two chakras gather in the uterus to provide the material basis for the growth of the embryo. Only under the condition that the kidney essence and kidney qi are sufficient and the yin and yang are balanced, the yin and yang of the uterus could rise and fall in an orderly manner and normal menstruation could occur. Menstruation stops during pregnancy when the blood gathers in the uterus to nourish the fetus and the kidneys have more important tasks to perform (nurturing the fetus). Using metal instruments to forcibly remove the pregnancy tissue during abortion is similar to “picking” unripe fruits and grains, which suddenly stops the pregnancy and results in damage to the kidneys, “violent damage to Chong-Ren” [9], and malfunction of Qi and blood. If the kidney qi is damaged, it is unable to push the blood to run normally, thus causing the blood to stagnate and become stagnant, resulting in abdominal pain, fever, leaking, and even infertility. Therefore, Chinese medicine should treat patients who undergo abortions by tonifying the kidneys and invigorating the blood. The timely application of Chinese herbal soup, Chinese patent medicine, and/or Chinese medicine special treatments (such as acupuncture, low-frequency electrical stimulation, ear acupuncture, acupuncture point application, and topical application of Chinese herbal medicine) after abortion could all play a role in tonifying the kidneys and activating the blood, helping to gradually restore the yuan-yin and yuan-yang in the kidneys, promoting the discharge of stagnant blood, and enabling the kidneys and uterus to resume their normal physiological functions and restore normal menstruation.

The low-frequency electrical stimulation treatment imitates the bioelectric pattern of the human body by placing electrodes on the skin surface and using low-frequency current pulses that are harmless to the human body to stimulate the acupuncture points. Its gentle treatment can be dosed or continuously stimulated without causing pain to the patient and is easily accepted by the patient. Its most prominent therapeutic effect is analgesia. Preoperative use of low-frequency acupoint electrical stimulation therapy in painless abortion was found to significantly improve the anesthetic effect, reduce the amount of anesthetic drugs, shorten the postoperative awakening time, and ensure surgical safety [10–12]. The implementation of low-frequency electrical stimulation therapy before and after abortion procedures could also reduce contraction pain in patients [12] and prevent the occurrence of abortion syndrome effectively [13]. Several studies have shown that low-frequency electrical stimulation therapy could effectively promote uterine rejuvenation and prevent uterine adhesion and other complications in patients who undergo abortion by improving endometrial blood perfusion. It could also improve endometrial receptivity, thus increasing the probability of repregnancy [14, 15]. In addition, it promotes oocyte development by increasing the number of high-quality follicles, improving ovulation rate, and promoting the return of menstruation [16].

In this study, the kidney-tonifying and blood-invigorating pills were made with reference to the Dingkun pills. As the prescription is confidential, the dosage of each drug is unclear. Hou et al. [17] conducted a pharmacodynamic study on the Dingkun pill and confirmed that it has no toxic side effects. It has significant effects on activating blood stasis, anti-inflammatory, and pain relief. Furthermore, it can promote endometrial growth. As the Dingkun pill has the strength of various prescriptions, it has a wide scope of treatment and can treat women's physical weakness and multiple diseases. Modern doctors have widely used it for treating polycystic ovary syndrome, postabortion, menorrhagia, premature ovarian failure, perimenopausal syndrome, postpartum uterine rejuvenation, and other diseases, all of which have achieved positive results. Studies have shown that the Dingkun pill can effectively increase the thickness of the endometrium and is effective in preventing secondary adhesions after surgery in patients with uterine adhesions. It could also significantly increase endogenous hormone levels, increase menstrual volume, and prolong menstruation [3]. It could relieve abdominal pain effectively, promote ovulation to improve menstrual irregularities, and enhance uterine repair, thus reducing postoperative complications [4].

HSP is present in almost all prokaryotic and eukaryotic organisms, and it has highly conserved properties. When an organism is exposed to stressors such as high temperature, tissue damage, ischemia, infection, and inflammation, the synthesis of such proteins is stimulated by heat to improve cell resistance, participate in synergistic immunity, regulate apoptosis, and maintain cell structure and function. On the basis of molecular weight, HSPs could be divided into the following families: HSP40, HSP60, HSP70, HSP90, and HSP110 families, and large- and small-molecule HSPs [18]. The HSP70 family is the most inducible and conserved class of the HSP family. Under normal circumstances, the expression of HSP70 is low, but under the stimulation of various unfavorable factors, it can induce cells to quickly translate and synthesize HSP70 to promote new protein synthesis, accelerate the repair of protein damage, and protect cells against harmful stimuli. It is more commonly expressed than other members of the HSP family. Human beings mainly synthesize HSP70 during the stress response, which participates in the development, differentiation, and activation of immune cells; participates in the growth and development of embryos; and regulates hormone secretion and other functional effects [19]. The HSP90 family is a class of steroid hormone-binding proteins, of which HSP90 is currently the most studied. Unlike other HSPs in the family, HSP90 is usually expressed at high levels right inside normal cells [20], and it is involved in the stabilization and activation of at least 300 proteins. HSP90 and its co-chaperones regulate important physiological processes, such as cell growth and survival, cell cycle regulation, hormone signaling, and apoptosis [21].

HSP70 and HSP90 are widely distributed in the endometrium and myometrium of normal women. HSP90 and HSP70 messenger RNAs (mRNAs) have been found to be localized in the myometrium, arterial smooth muscle, and endometrial glandular epithelial cells. High expression of both was hypothesized to be caused by an increase in estrogen receptor mRNA and protein, which, in turn, increased the action of estrogen on the myometrium, thus enhancing uterine sensitivity and favoring uterine contraction [22]. In the present study, the serum concentrations of HSP90 and HSP70 were elevated in both groups at 2 weeks postoperatively compared with those preoperatively, and this finding may be related to the above mechanism. Two weeks after the operation, the serum concentrations of HSP90 and HSP70 were higher in the control patients than in the experimental group, but an ultrasound review indicated that uterine contractions were better in the experimental group than in the control group. This finding may be due to the fact that dual treatment with low-frequency electrical stimulation and kidney-reinforcing pills reduced the synthetic secretion of HSP70 and HSP90 in the experimental group. Thus, this treatment protocol is more beneficial in promoting uterine contraction after abortion. HSP70 and HSP90 were found to be closely associated with endometrial proliferation and secretion [23]. Furthermore, they are associated with the growth and functional maintenance of the normal-state endometrium [24–26]. In the present study, both groups were reviewed with ultrasound at 2 weeks postoperatively, and the results showed that the endometrial thickness was better in the experimental group than in the control group. The results showed that the time of the first menstrual recovery and menstrual volume were more satisfactory in the experimental group than in the control group, and this finding may be related to the repair of endometrial hyperplasia by HSP70 and HSP90. Therefore, low-frequency electrical stimulation combined with kidney activation pills is more beneficial to accelerate uterine contraction and endometrial hyperplasia repair, which is conducive to promoting menstrual resumption and maintaining menstrual flow.

When miscarriage occurs, the levels of HSP90 and HSP70 also change physically, and Peng et al. [27] found that apoptosis could lead to spontaneous abortion, along with a significant increase in HSP70 expression in metaphase tissue. Although HSP70 does not inhibit apoptosis, it has a protective effect on metaphase tissue. Overexpression of HSP70 was also suggested to be detrimental to pregnancy [28]. Matsuda et al. [29] found that women with unexplained RPL (recurrent pregnancy loss) had significantly higher levels of anti-HSP70 antibodies than normal pregnant women. Gulic et al. [30] found a strong positive expression of HSP90 in the placental meconium of patients with auditory abortion compared with that in normal early pregnant women. Later, they found lower levels of HSP70 and interleukin-15 (IL-15) in the meconium of patients with preeclampsia than in those with normal pregnancy and suggested that the downregulation of HSP70 and IL-15 levels supports the retention of pregnancy material in patients with auditory abortion. This finding provides a diagnostic basis for abortion failure. Lu [20] found that the expression levels of HSP70 and HSP90 were increased in the meconium tissues of both patients who medically aborted and induced abortion; higher in medically aborted than in induced abortions; and with a positive correlation with bleeding time. The results of this study showed that the postoperative serum HSP70 and HSP90 concentrations were significantly higher in both groups than in the preoperative period, consistent with the findings of the present study. Thus, the high expression of HSP70 and HSP90 after abortion may have a protective effect on the uterus and endometrium. In addition, the postoperative serum HSP70 and HSP90 concentrations of patients in the experimental group were significantly lower than those in the control group, indicating that low-frequency electrical stimulation combined with tonification of kidney and blood pills was more protective than low-frequency electrical stimulation therapy separately.

The results of this study showed that the patients in the experimental group had less postoperative abdominal pain than the control group, and the duration was shorter. This result indicated that low-frequency electrical stimulation combined with tonifying kidney and blood pills is more effective in improving postoperative abdominal pain in patients who underwent abortion. Although no significant difference was found in the amount of postoperative vaginal bleeding, duration of bleeding, uterine residue, uterine blood accumulation, and urine pregnancy test results between the two groups, the results from the literature showed that tonifying the kidney and invigorating blood pills could effectively promote uterine rejuvenation in patients after abortion. In addition, the results of the present study showed that the patients in the experimental group had a longer period of the first menstrual resumption than those in the control group, and such findings may be caused by the blood-activating effect of herbs. Neither the experimental group nor the control group experienced any side effects during the treatment follow-up period, thereby indicating the safety of this study.

This study confirmed that low-frequency electrical stimulation combined with tonifying kidney and blood pills could effectively promote uterine rejuvenation after painless abortion, which is conducive to improving patients' postoperative abdominal pain, promoting the return of menstruation, and maintaining menstrual flow. For ethical reasons, a blank control group was not established. Thus, no observation could be performed as to whether the HSP70 and HSP90 concentrations could be reduced or increased after treatment. Animal experiments could be conducted in the future. Because of the short study period and unstable patient adherence, long-termfollow-up observation of all cases was not achieved in this study, making it difficult to understand the long-term efficacy and complications. The follow-up period of patients after abortion could be extended in the follow-up study to fully explore the possibility of Chinese medicine to prevent and treat various near- and long-term complications (such as menstrual disorders, secondary infertility, and recurrent miscarriage) after abortion.

## Figures and Tables

**Table 1:** Comparison of the degree of postoperative abdominal pain.

Group	*n*	The degree of abdominal pain				*x * ^2^ value	*p* value
		None	Light	Medium	Severe		
Observation group	202	158 (78.2%)	31 (15.3%)	12 (5.9%)	1 (0.5%)	4.458	0.035
Control group	119	80 (67.2%)	27 (22.7%)	10 (8.4%)	2 (1.7%)		

Note: Degree of abdominal pain (none means no abdominal pain; mild means occasional cramping/swelling/stabbing pain in small clothes; medium means frequent episodes of the above symptoms; severe means persistent episodes of the above symptoms).

**Table 2:** Comparison of the duration of abdominal pain and vaginal bleeding (days).

Observed indicators	Observation group (*n* = 202)	Control group (*n* = 119)	*t* value	*p* value
Duration of abdominal pain (days)	2.74 ± 0.69	4.31 ± 0.92	−15.999	0.000
Duration of bleeding (days)	7.92 ± 4.82	9.82 ± 5.33	−3.277	0.822

**Table 3:** Comparison of the volume of vaginal bleeding (mL).

Group	*n*	Bleeding volume (ml)			*x * ^2^ value	*p* value
		<20	20–60	>60		
Observation group	202	160 (79.2%)	34 (16.8%)	8 (4.0%)	2.053	0.358
Control group	119	89 (74.8%)	21 (17.6%)	9 (7.6%)		

**Table 4:** Comparison of uterine size and endometrial thickness at 2-week postoperative follow-up ultrasound.

Observed indicators	Observation group (*n* = 202)	Control group (*n* = 119)	*t* value	*p* value
Endometrial thickness (mm)	6.15 ± 3.54	4.25 ± 2.31	5.791	0.000
Uterine size (cm)	11.33 ± 0.69	14.93 ± 0.79	−41.226	0.000

**Table 5:** Comparison of residual and accumulated blood in the uterine cavity.

Observed indicators	Observation group (*n* = 202)	Control group (*n* = 119)	*t* value	*p* value
Uterine residual (yes/no)	33/169	18/101	0.082	0.774
Uterine effusion (yes/no)	40/162	28/91	0.623	0.430

**Table 6:** Comparison of urine pregnancy test results at 2 weeks postoperation.

Group	*n*	Results of urine pregnancy test			*x * ^2^ value	*p* value
		Negative	Weakly positive	Positive		
Observation group	202	165 (81.7%)	11 (5.4%)	26 (12.9%)	0.280	0.869
Control group	119	98 (81.5%)	8 (6.7%)	13 (11.8%)		

**Table 7:** Preoperative and postoperative serum HSP70 and 90 concentrations (pg/ml) in each group.

Observed indicators	HSP70		*t* value	*p* value	HSP90		*t* value	*p* value
	Pre-op	Post-op			Pre-op	Post-op		
Observation group	47.01 ± 14.42	88.56 ± 25.68	−19.066	0.000	72.24 ± 19.085	106.96 ± 34.27	−12.768	0.000
Control group	48.42 ± 13.61	107.2 ± 22.90	−22.813	0.000	76.13 ± 17.138	121.76 ± 36.48	−12.212	0.000
*T* value	−0.861	−6.555			−1.885	7.247		
*p* value	0.390	0.000			0.060	0.000		

**Table 8:** Comparison of time of menstrual return and period.

Observed indicators	Observation group (*n* = 202)	Control group (*n* = 119)	*t* value	*p* value
Time of menstrual resumption (days)	38.72 ± 8.13	42.54 ± 8.64	−3.693	0.000
Period of resumption (days)	6.04 ± 1.64	5.31 ± 1.48	3.979	0.000

**Table 9:** Comparison between the return period and the original period.

Group	*n*	Menstrual period		*t* value	*p* value
		Original period (days)	Period of resumption (days)		
Observation group	202	5.55 ± 1.29	6.04 ± 1.64	−3.128	0.002
Control group	119	5.47 ± 1.39	5.31 ± 1.48	0.856	0.393

**Table 10:** Comparison of menstrual volume at resumption.

Group	*n*	Menstrual volume at resumption			*x * ^2^ value	*p* value
		Same as pre-op	Decreased	Increased		
Observation group	202	155 (76.7%)	21 (10.4%)	26 (12.9%)	25.532	0.000
Control group	119	72 (60.5%)	39 (32.8%)	8 (6.7%)		

## Data Availability

The labeled dataset used to support the findings of this study are available from the corresponding author upon request.
